# Comparison of different blood pressure indices for the prediction of prevalent diabetic nephropathy in a sub-Saharan African population with type 2 diabetes

**Published:** 2012-04-11

**Authors:** Simeon-Pierre Choukem, Anastase Dzudie, Mesmin Dehayem, Marie-Patrice Halle, Marie-Solange Doualla, Henry Luma, Andre-Pascal Kengne

**Affiliations:** 1 Department of Internal Medicine, Douala General Hospital, Douala, Cameroon; 2 Department of Clinical Sciences, Faculty of Health Science, University of Buea, Buea, Cameroon; 3 Diabetes and Endocrine Unit and National Obesity Centre, Yaounde Central Hospital, Yaounde, Cameroon; 4 Department of Internal Medicine and Subspecialties, Faculty of Medicine and Biomedical Science, University of Yaounde I, Yaounde, Cameroon; 5 Department of Medicine, Faculty of Health Science, University of Cape Town & Medical Research Council, Cape Town, South Africa

**Keywords:** Blood pressure, diabetes mellitus, discrimination, nephropathy, sub-Saharan Africans

## Abstract

**Background:**

The association between blood pressure (BP) and diabetic kidney diseases in Africans has been less well investigated. We assessed and compared the strength of the association and discriminatory capability of systolic (SBP), diastolic (DBP) BP, pulse pressure (PP) and mean arterial blood pressure (MAP) for nephropathy risk in sub-Saharan Africans with type 2 diabetes.

**Methods:**

Participants were 420 consecutive individuals (49% men) with type 2 diabetes receiving chronic care in two main referral centres in the two major cities of Cameroon. Logistic regression models were used to compute the odd ratio (OR) and 95% confidence interval (95% CI) for a standard deviation (SD) higher level of SBP (25 mmHg), DBP (13), PP (18) and MAP (16) with nephropathy risk. Discrimination was assessed and compared with c-statistics and relative integrated discrimination improvement (RIDI, %).

**Results:**

The adjusted OR (95% CI) for nephropathy with each SD higher BP variable were: 1.45 (1.15-1.84) for SBP, 1.33 (1.06-1.66) for DBP, 1.35 (1.06-1.71) for PP and 1.42 (1.13-1.78) for MAP. C-statistic comparison showed no difference in discrimination of models with each of the BP variables (p-values ≥ 0.69 for c-statistics comparison). However, RIDI statistic always showed and enhancement in models discrimination when other BP variables were replaced with SBP, although such an enhancement was marginal for MAP. Using BP combination modestly improved models? discrimination.

**Conclusion:**

SBP was the best predictor of prevalent nephropathy in this population, while DBP was the less effective. This may have implication for kidney disease risk stratification and protection.

## Background

Diabetic nephropathy remains the leading cause of end stage renal disease worldwide and is associated with increased morbidity, mortality and health costs [1, 2]. The importance of diabetic kidney diseases is expected to increase even further as the result of the growing population of individuals with diabetes, particularly in developing countries [3]. Diabetic nephropathy is the result of the interplay between several factors specific or non specific of diabetes such as hypertension and non-optimal blood pressure levels [1, 2].

The relationship between blood pressure (BP) and the risk and progression of diabetic nephropathy is well established [4, 5], and lowering BP is a major target of strategies for preventing or slowing the progression of renal involvements in diabetes [6]. These strategies are usually based on cut-offs of BP indices, and systolic and diastolic BP in particular. This approach fails to capture the continuous association between blood pressure and risks of nephropathy, but also inherently assigns equal importance to different BP indices. Previous studies mainly based on Caucasian populations have evaluated the impact of ambulatory BP or circadian BP variations on the development and progression of nephropathy in type 2 diabetes [7–11]. However, whether different BP indices are similar determinants of the risk of diabetic nephropathy has been less investigated. In the RENAAL study population, the effects of SBP, DBP and pulse pressure on various renal outcomes were examined [5]. Findings were conclusive of a strong role of SBP and pulse pressure, but not DBP as predictors of progression of nephropathy. Furthermore, to the best of our knowledge no study has examined the relative importance of BP for predicting diabetic nephropathy in African populations. Addressing these issues has relevance for the improvement of risk evaluation and risk reduction strategies in the routine care of individuals with diabetes.

The aim of the present study was to assess and compare the strength and discriminatory power of different BP indices in predicting prevalent nephropathy among sub-Saharan African individuals with type 2 diabetes.

## Methods

### Study population

Participants in this cross-sectional study were Cameroonian men and women with various known duration of doctor-diagnosed type 2 diabetes. They were consecutively recruited from the out-patient sections of the Yaoundé Central Hospital and Douala General Hospital diabetes units, the two major ones in Cameroon. Specialized chronic diabetes care in these two units is provided by diabetologists. From 2006 (in Yaoundé) and 2008 (in Douala), individuals with diabetes receiving care in these settings are required to have an annual check-up. This includes clinical examination, biological investigations, eye examination and a resting electrocardiogram. Recruitment for this study was restricted to participants who had at least one annual check-up between January 2008 and October 2010. Participants were included retrospectively from January 2008 to December 2009, and prospectively thereafter. The study was approved by the Cameroon National Ethic Committee.

### Evaluation

For each eligible participant, data were collected on medical and family history, and behavioural factors (smoking). Physical measurements were height, weight, blood pressure, heart rate, and waist and hip circumferences, assessed following standardized methods. Calculations included mean arterial pressure (DBP+1/3(SBP-DBP)), pulse pressure (SBP-DBP) and body mass index (weight(kg)/(height(m))^2^).

### Diagnosis of nephropathy

For every patients, urine protein was screened in non-timed middle stream urine samples with the use of a dipstick (Combur^10^ Test^®^, Roche Diagnostics GmbH, Mannheim, Germany), and was considered positive only when confirmed by a second sample, and after elimination of confounders including fever, glycosuria, hematuria, urinary tract infection, and exercise prior to test. Serum creatinine was measured in all participants using routine laboratory techniques. Diabetic nephropathy was defined as the presence of positive proteinuria with or without low creatinine clearance (< 90ml per min per 1.73mm^3^ of body surface) using the Cockroft-Gault equation.

### Statistical analysis

Data were analyzed with the use of SAS/STAT v.9.1 for Windows^®^ (SAS Institute Inc., Cary, NC, USA). Comparison of variables used chi squared test and variants for qualitative variables and Student t test or non-parametric equivalents for continuous variables. Results are presented as count (percentages), mean and standard deviation (SD) or median and interquartile range as appropriate.

Logistic regressions models were used to assess the independent association between prevalent nephropathy and each blood pressure index. The independent association was assessed in two ways: first by fitting a continuous predictor to obtain the odd ratio (OR) and the 95% confidence interval (95% CI) for a standard deviation higher level of pressure variables and, secondly by comparing the association across fourths of blood pressure variables. In the later analyses, the lower fourth of BP variables was always used as reference, and 95% CI were computed with the use of the floating absolute risk methods [12]. This approach allows for the computation of the CI for the referent category, and also allows for mutual comparison of non-referent categories. This is not possible using conventional approaches. Log-linearity of the associations between each BP variable and prevalent nephropathy was explored. All models were adjusted for age, sex, recruitment centre and treated hypertension.

The ability of BP variables to discriminate between participants who had and those who did not have nephropathy was assessed using area under the receiver operating characteristic curves (AUC) and the relative integrated discrimination improvement (RIDI) which measures the percentage increased discrimination when an extra variable is added to a prediction model [13, 14]. AUC comparisons were examined with nonparametric methods [15]. Bootstrap techniques were used to derive the 95% CI for the RIDI estimates, which were based on 1000 replications. The likelihood ratio c^2^ statistics for each event category were calculated by comparing multivariate regression models with and without a single BP variable to assess improvement in model fit. Secondary analyses were conducted testing for the combination of BP variables (SBP+DBP or MAP+PP) and SBP^2^, DBP^2^, PP^2^ or MAP^2^ in logistic regression models.

## Results

The profile of the 420 men and women with type 2 diabetes is described in Table 1. Compared with women, men were more likely to be current smokers (13.1% vs. 2.3%, p<0.001), to have a high waist-to-hip ratio (0.96 vs. 0.91, p<0.001), and less likely to have a high body mass index (27.2 vs. 29.7 kg/m^2^, p<0.001) and to be on beta blockers (3.4% vs. 10.8%, p=0.004). The Pearson correlation coefficients for blood pressure variables were: 0.72 (SBP vs. DBP), 0.86 (SBP vs. PP), 0.92 (SBP vs. MAP), 0.27 (DBP vs. PP), 0.93 (DBP vs. MAP) and 0.61 (PP vs. MAP). The prevalence of nephropathy was 31% overall, 32% in men and 30% in women (p=0.68).

**Table 1:** Profile of the 420 men and women with type 2 diabetes

Variables	Men	Women	p	Total
	207 (49%)	213 (51%)		420
Age, years	55.9 (9.8)	57.5 (10)	0.09	56.7 (9.9)
Median (range) known duration of diabetes, years	4 (0-9)	4 (1-8)	0.71	4 (1-9)
Parental history of diabetes, n (%)	103 (49.7%)	110 (51.6%)	0.69	213 (50.7%)
Smoking, n (%)	27 (13.1%)	5 (2.3%)	<0.001	32(7.6%)
Body mass index, Kg/m^2^	27.2 (4)	29.7 (6)	<0.001	28.5 (5.2)
Waist circumference, cm	95.3 (10.8)	94.9 (12.9)	0.71	95.1 (11.9)
Waist-to-hip ratio	0.96 (0.08)	0.91 (0.11)	<0.001	0.94 (0.10)
**Hypertension and treatments**				
Systolic blood pressure, mmHg	142.8 (23.6)	141.6 (26.9)	0.61	142.2 (25.3)
Diastolic blood pressure, mmHg	85.6 (12.2)	84.5 (14.2)	0.37	85.1 (13.2)
Pulse pressure, mmHg	57.2 (16.8)	57.1 (19.5)	0.95	57.1 (18.2)
Hypertension, n (%)	97 (46.8%)	114 (53.5%)	0.17	211 (50.2%)
Any blood pressure lowering medication	83 (40.1%)	103 (48.4%)	0.09	186 (44.3%)
ACE inhibitors	70 (33.8%)	69 (32.4%)	0.84	139 (33.1%)
ARA II antagonists	2 (1%)	3 (1.4%)	1	5 (1.2%)
Diuretics	54 (26.1%)	64 (30%)	0.37	118 (28.1%)
Calcium chanel blockers	33 (15.9%)	36 (16.9%)	0.79	69 (16.4%)
Beta blockers	7 (3.4%)	23 (10.8%)	0.004	30 (7.1%)
Median (range) creatinine clearance, ml/min/1.73m^2^	91 (70-113)	88 (63-108)	0.23	89 (67-111)
Any diabetic nephropathy, n (%)	64 (32%)	66 (30%)	0.68	130 (31%)

**Figure 1:**
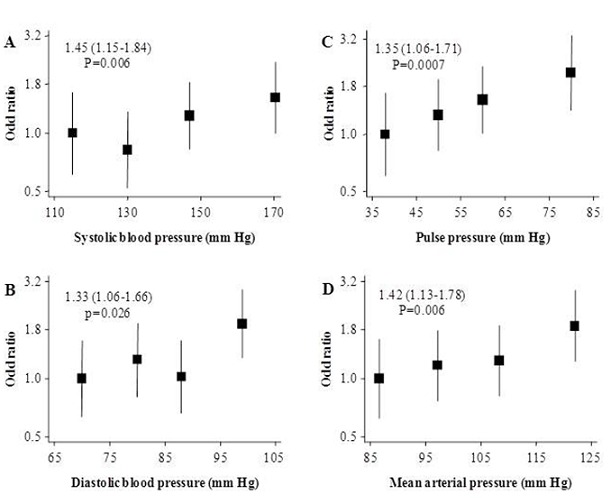
Adjusted odd ratios and 95% confidence interval (95% CI) for the presence of nephropathy comparing the fourths for systolic (panel A), diastolic (panel B), pulse (panel C) and mean (panel D) arterial blood pressure. All analyses were adjusted by age, sex, use of blood pressure lowering medication and recruitment centres. Boxes are for the point estimates (odd ratios) and the vertical bars about represent the 95% confidence intervals. The size of the box is proportional to the inverse variance of the natural logarithm of the odd ratio, and boxes are plotted against the median specific to each fourth of blood pressure variable on the horizontal axis. The vertical axis is on log-scale to allow a balanced distribution of the 95% CI about the effect estimates, and a better visualisation of the trends of the associations.

There was a continuous and positive association between blood pressure indices and nephropathy. For instance a standard deviation higher level of BP variables was associated with 45% (95% CI: 15-84) risk of nephropathy for systolic blood pressure, 33% (6-66) for diastolic blood pressure, 35% (6-71%) for pulse pressure and 42% (13-78) for mean arterial pressure. There was also a graded association between BP and nephropathy across fourths of BP indices (Figure 1). This association was linear (all p?0.03 for linear trend), with however some indication that the association could be non-linear for diastolic pressure (p=0.09 for non-linearity).

When models with BP variables and covariates were compared with models with co-variates only, the difference in likelihood-ratio X^2^ tests was in favor of a stronger association between SBP and nephropathy, although the difference with MAP was marginal (Table 2). There was no significant interaction between BP variables and sex (all p≤0.17) and use of blood pressure lowering treatment (all p≤0.51) for the risk of nephropathy.

**Table 2:** Area under the receiver operating characteristics curves (AUC) and 95% confidence interval, Akaike information criterion (AIC), difference in likelihood ratio chi square (with model with covariates only) and p-value, and calibration chi square and p-value for the prediction of diabetic nephropathy

Model	AUC	AIC	Delta likelihood ratio c^2^	Calibration c^2^ (p-value)
SBP (A)	0.620 (0.562-0.678)	511.871	9.946	8.92 (0.35)
DBP (B)	0.618 (0.560-0.677)	515.376	6.442	7.86 (0.45)
PP (C)	0.615 (0.558-0.673)	515.772	6.046	5.24 (0.73)
MAP (D)	0.622 (0.564-0.680)	512.564	9.254	5.44 (0.71)
SBP + DBP (E)	0.621 (0.563-0.679)	513.749	10.069	6.32 (0.61)
PP + MAP (F)	0.621 (0.563-0.679)	513.749	10.069	6.32 (0.61)

Models are adjusted for age, sex, use of blood pressure lowering medication and recruitment centre. P value for differences in AUC: A-B (0.91), A-C (0.69), A-D (0.84), A-E (0.78), A-F (0.78) B-C (0.89), B-D (0.67), B-E (0.86), B-F (0.86), C-D (0.72), C-E (0.69), C-F (0.69), D-E (0.88), D-F (0.88), E-F (1); Overall p-value (0.93) for the difference in AUC for all the models above. Systolic blood pressure (SBP), diastolic blood pressure (DBP), pulse pressure (PP) and mean arterial blood pressure (MAP)

Based on Akaïke's information criterion (AIC) comparison, models with SBP was the best performing and model with DBP the least. Models combining BP variables (SBP+DBP, PP+MAP) did less well than model with MAP alone (Table 2). However, the discriminatory capability of models, as appreciated by the AUC ROC was not appreciably different between models with single or combination of BP variables (all p≤0.69 for AUC comparison (Table 2). Using the RIDI statistic, models with SBP and MAP were significantly better than model with either DBP or PP alone. There was a further deterioration of 2.5% (95% CI: 1.9-3.2%) when SBP was replaced by MAP in models. Models with combination of BP variables did better than model with single BP variable, but such improvement although significant was small when compared with model with either BP variable alone (Table 3). Based on calibration X^2^ statistics, the best fit of the data was obtained with model with PP (Table 2). Adding the quadratic terms of BP variables (SBP^2^, DBP^2^, PP^2^ or MAP^2^) to model that already included relevant BP variable did not improve model performance (data not shown).

**Table 3:** Relative integrated discrimination improvement statistics (RIDI, %) statistic comparing models with a given blood pressure variable with models for which the variable has been replaced by another blood pressure variable or their combination.

Model	DBP	PP	MAP	SBP+DBP	PP+MAP
**SBP**	-13.7 (-14.8 to -12.6)	-16.0 (-16.9 to -15.1)	-2.5 (-3.2 to -1.9)	5.1 (4.6 to 5.6)	5.1 ( 4.6 to 5.6)
**DBP**	NA	3.8 (1.4 to 6.2)	15.4 (14.6 to 16.3)	26.9 (24.9 to 28.8)	15.4 (24.6 to 16.3)
**PP**		NA	21.9 (19.5 to 24.2)	31.1 (28.0 to 32.2)	31.1 (28.0 to 32.2)
**MAP**			NA	8.9 (8.1 to 9.7)	8.9 (8.1 to 9.7)
**SBP+DBP**				NA	0

Systolic blood pressure(SBP), diastolic blood pressure (DBP), pulse pressure (PP) and mean arterial blood pressure (MAP)

## Discussion

The current analyses suggest that blood pressure indices including SBP, DBP, PP and MAP are continuously associated with the risk of prevalent diabetic nephropathy. These associations appear to be log-linear, with a SD higher level of each pressure variable contributing the same range of effect sizes as indicated by the overlapping confidence interval about the effect estimates. However, the association with SBP was stronger than those with other BP indices based on likelihood ratio comparison. Models with each BP index had similar discriminatory power for prevalent nephropathy based on AUC comparisons. However, the RIDI statistics were in favor of a significant advantage of models with SBP, and to some extend models with MAP. Combining BP indices only marginally improved discrimination when compared with models with single BP variable.

Globally, the risks of kidney diseases afforded by blood pressure have been investigated for prevalent-, incident kidney diseases, and for the progression of established disease. The focus has often been on people with non-optimal blood pressures. Risks have also been assessed across the continuum of BP measurements including in people with diabetes. Comparisons have often examined the issues of whether ambulatory, office, daytime or night-time blood pressures were similar determinants of nephropathies [16–19]. Few studies have directly compared different BP variables from the same measurements for the prediction of nephropathy, including in people with diabetes. These studies have generally emphasized the importance of SBP over DBP in promoting the development and progression of diabetic nephropathy [20]. The relationship with MAP has been less tested, and whether PP is superior to SBP as a predictor of nephropathy is still debated, owing in part to the variable associations between different PP measurements and renal outcomes. In the Ohasama Study [16], SBP was a significant predictor of microalbuminuria while PP was not. In a cohort of London individuals with chronic kidney disease at baseline, pulse pressure was stronger than systolic blood pressure in predicting further decline in kidney function over a mean follow-up duration of 172 days [21]. This was in keeping with findings from the Bordeaux cohort study of individuals with essential hypertension in which pulse pressure emerged as a significant predictor of the decline in kidney function [22]. In that study, pulse pressure was examined in the same Cox models with SBP and DBP. In such a model however, the DBP will cancel out as the result of introducing PP (the difference SBP-DBP) leaving SBP as the sole BP variable in the model. The reported results may therefore reflect more the effect of SBP. In the Informatics for Diabetes Education and Telemedicine (IDEATel) cohort of older individuals with type 2 diabetes, ambulatory 24-hour PP, but not office PP, was an independent determinant of the progression of albuminuria [18]. In post-hoc analyses from the RENAAL trial, SBP and PP were similar determinants of the risk of end-stage renal disease while DBP was not associated with the outcomes [5]. Comparisons in this study however were based on the size of risk estimates (hazard ratio) associated with a 10 mmHg higher level of each BP index, which is a non optimal approach for the intended purpose.

Unlike DBP and PP, we are not aware of a study that has directly compared MAP and SBP or other pressure variables for renal outcomes in people with diabetes. In the Baltimore Longitudinal Study of Ageing (1% with diabetes) [23], both cross-sectional and longitudinal components of the four BP indices were examined in relation with urinary albumin excretion (UAE). None of the BP variables was associated with UAE in women. In men, only SBP and PP were associated with UAE while DBP and MAP were not, with further suggestion that PP was better than SBP. Comparisons however were based on nested models and would best reflect the effects combination of BP indices than single variable [23]. The finding of a stronger association between MAP and diabetic nephropathy risk as compared with that for PP may therefore be a new finding in our study. Because BP, a promoter of kidney disease is further modified once the disease is established, it is in major ways difficult to provide an accurate interpretation to some findings from our study, given its cross-sectional nature.

The current study has some limitations. Our analyses were based on a relatively small number of outcome (130 prevalent nephropathies), and accordingly a limited power to reliably examine some issues. For instance, our assessment of the linearity of the associations was based on four subgroups, and therefore less likely to detect departures from linearity. The cross-sectional nature of the study makes any inference about causality less reliable. Also, in the absence of a quantitative assessment of urinary albumin excretion including microalbuminuria, we couldn't examine the association between BP indices and our outcome of interest across the continuum of urinary albumin excretion. Our study also has major advantages. These include the first examination of this important research question in African populations, and the use of more advanced and sensitive statistical methods to reliably assess and compare the discriminatory capabilities of BP variables and derived predictions models.

## Conclusion

In conclusion, although different BP indices are significant predictors of prevalent diabetic nephropathy, the present study suggests that SBP is the more effective predictor, and combination of BP variables adds little to the discrimination of nephropathy beyond SBP. These findings largely support applicability in this population of current recommendations for preventing the onset and slowing the progression of diabetic nephropathy that emphasize the importance of systolic blood pressure.
